# Supplementary figures and images for: RNA-seq gene expression profiling of the bladder in a mouse model of urogenital schistosomiasis
Source: bioRxiv. 2024 Jun 29:2024.06.29.601185. Preprint. [Version 1] doi: 10.1101/2024.06.29.601185 (PMC11230422; doi:10.1101/2024.06.29.601185)

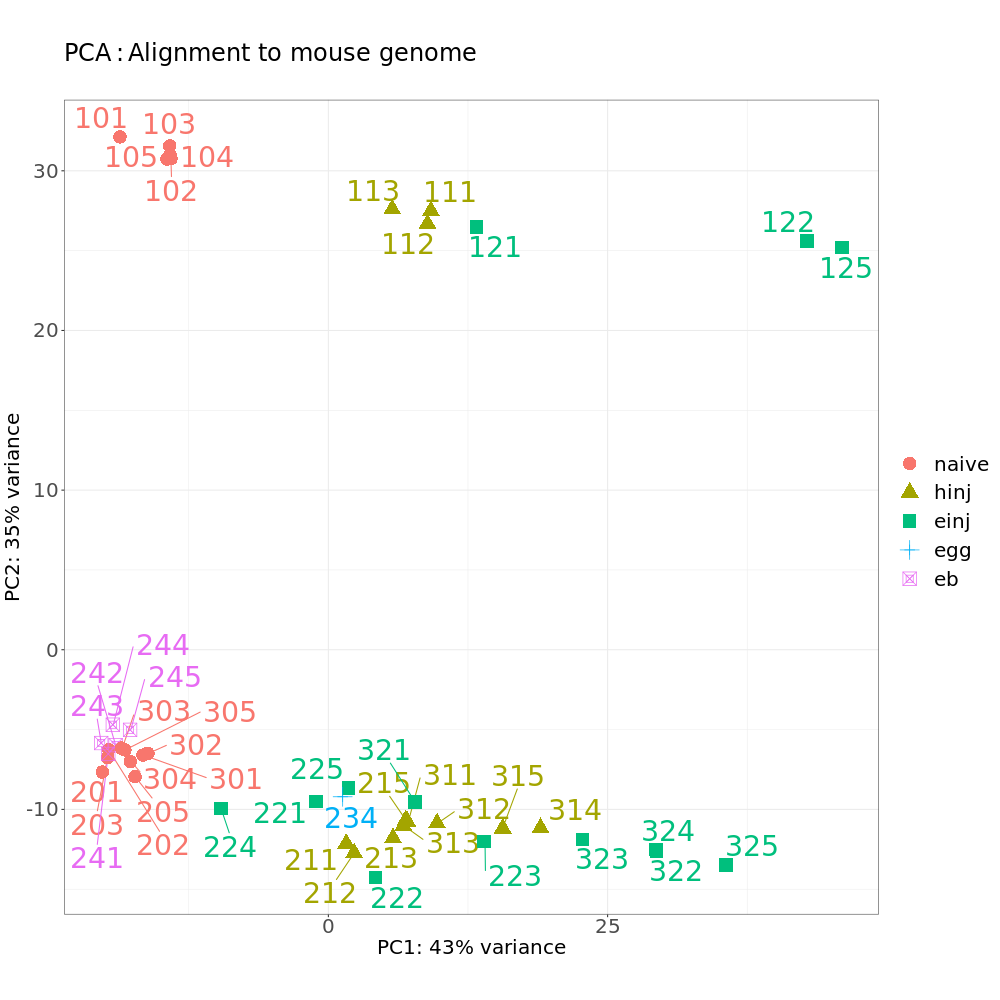

Supplement: Supplement 6 — [z06_mm-pca.png] Supplementary 6. Principal component analysis of samples from three independent sets of experiments aligned to the mouse genome. Several egg-injected samples (121, 221-225, 321) did not receive eggs because of technical error and cluster with the vehicle-injected samples. Subsequent analyses consider samples from two sets of experiments (2xx and 3xx). Key: naive, no injection (soft-red filled circles); hinj, vehicle-injected (olive-yellow filled triangles); einj, S. haematobium egg-injected (cyan-green filled squares); egg, S. haematobium egg alone (blue crosses); eb, S. haematobium egg mixed with bladder tissue from mice that did not undergo bladder wall injection (soft-magenta open squares). [file media-6.tif]

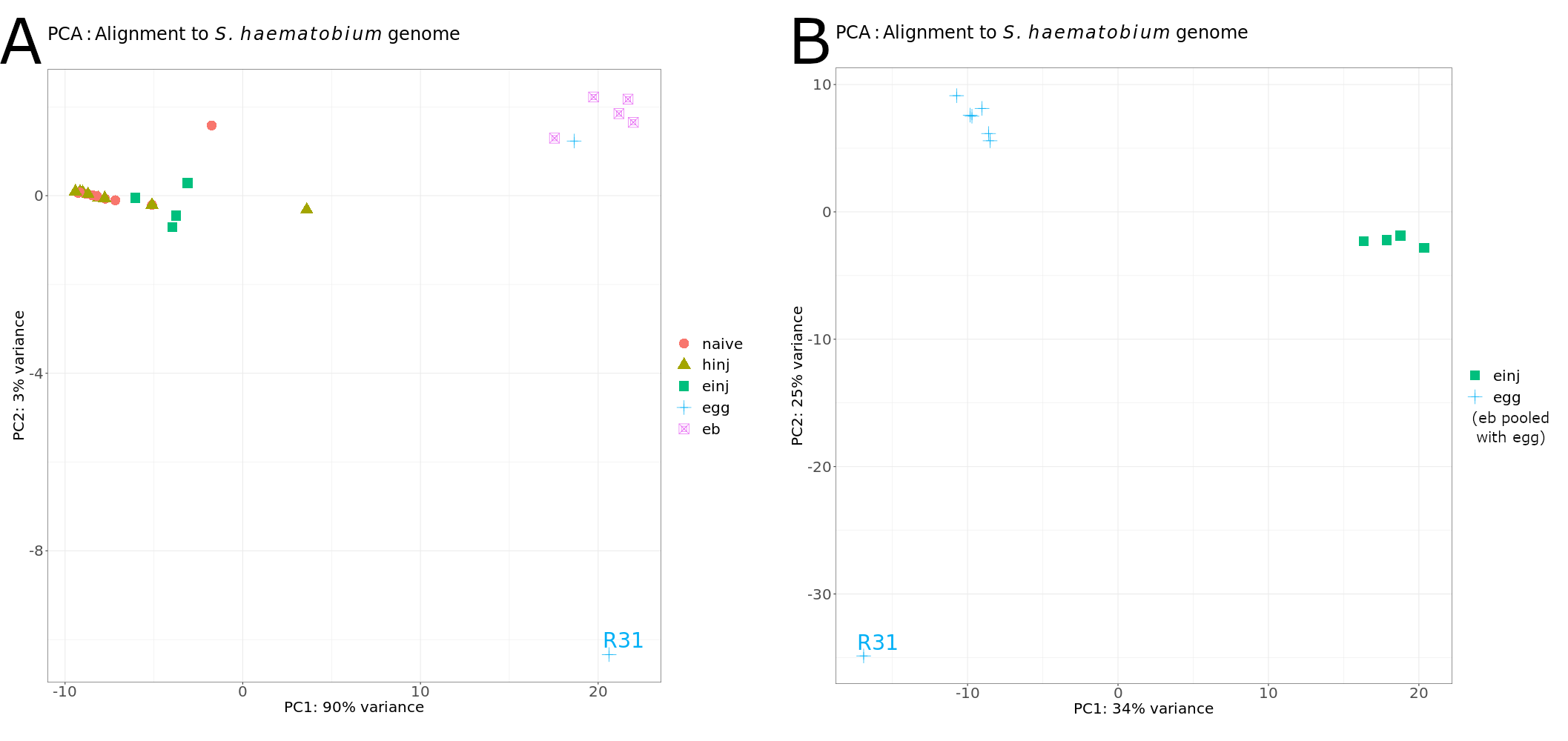

Supplement: Supplement 13 — [z13_sh_pca_r31.tif] Supplementary 13. Principal component analysis of samples from two independent sets of experiments aligned to the S. haematobium genome including egg reference data. Key: naive, no injection (soft-red filled circles); hinj, vehicle-injected (olive-yellow filled triangles); einj, S. haematobium egg-injected (cyan-green filled squares); egg, S. haematobium egg alone (blue crosses); eb, S. haematobium egg mixed with bladder tissue from mice that did not undergo bladder wall injection (soft-magenta open squares). A) all sample groups. B) egg-injected (einj), egg-alone (egg), and egg mixed with bladder tissue from mice that did not undergo injection (eb); eb samples are pooled together with egg samples. [file media-13.tif]
